# Quality of knee strengthening exercises performed at home deteriorates after one week

**DOI:** 10.1186/s12891-022-05120-3

**Published:** 2022-02-19

**Authors:** Ulrike H. Mitchell, Hyunwook Lee, Hayden E. Dennis, Matthew K. Seeley

**Affiliations:** Department of Exercise Sciences, Brigham Young University, 106 SFH, Provo, UT USA

**Keywords:** Physical therapy exercises, Exercise retention, Motion capture system

## Abstract

**Background:**

To compare the performance (as determined by lower extremity kinematics) of knee exercises in healthy middle-aged and older individuals immediately after instruction and one week later.

**Methods:**

This is a cross-sectional study in a laboratory setting. Nineteen healthy volunteers (age [y] 63.1 ± 8.6, mass [kg] 76.3 ± 14.7, height [m] 1.7 ± 0.1) participated in this study. High speed video and reflective markers were used to track motion during four exercises. The exercises were knee flexion, straight leg raise, and “V “in supine position, and hip abduction in side lying position. All participants received verbal and tactile cues during the training phase and the therapist observed and, if necessary, corrected the exercises. Upon return a week later the participants performed the same exercises without any further instructions. Knee and hip sagittal and rotational angles were extracted from the motion capture. A repeated measures t-test was used to compare the motions between two visits.

**Results:**

Participants demonstrated more knee flexion during straight leg raise and “V in” exercises at the 2nd visit compared to the 1st visit (both *p* <  0.05). During the “V out” exercise, they performed more external rotation (*p* <  0.05) while they showed more internal rotation during the “V in” exercise at the 2nd visit compared to the 1st visit.

**Conclusions:**

Exercise performance declined significantly in healthy middle-aged and older individuals one week after instruction. This decline occurred despite an instructional exercise sheet being given to every participant. Other approaches designed to help individuals retain the ability to perform rehabilitative exercises correctly need to be explored.

## Background

The prevalence of osteoarthritis increases with age and has been reported to lie between 19.2% [1] and 27.8% [2] in people of 45 years and older and at 37.4% [3] in people 60 years and older. It mostly affects load bearing, larger joints, like the hips and knees and the small joints of the hands [4]. Exercises that promote range of motion and strength have been recommended for the prevention and treatment of associated symptoms, such as decreases in quality of life and pain [5, 6]. A large study in Denmark [7] showed that a 6-week physical therapy program, consisting of patient education and exercises, improved physical function and decreased pain, medication intake, and sick leave time. Other disorders [8] and most post-surgery knee conditions, such as total knee arthroplasty [9], require range of motion and strengthening exercises to the legs. Unfortunately, due to inconvenient logistics, non-availability of physical therapy, patient’s lack of insurance and other factors, many patients cannot or do not take advantage of similar services. In those cases, patients are sent home with exercise instructions.

The effectiveness of home exercises is controversial. For example, several studies (e.g., [10, 11]) have shown that direct supervision from a physical therapist may not be required for effective physical therapy; these studies reported no difference in outcomes between patients after total knee arthroplasty who received physical therapy in an outpatient clinic and those who performed exercises at home. Other studies have shown that the effectiveness of home exercises is at least partially dependent on the patient’s compliance and adherence to the protocol. Non-compliance to exercise programs affects the effectiveness of the intervention [12], might prolong the rehabilitation time unnecessarily, increases the risk of recurrent injury or flare-ups [13] and increase levels of pain and impairment [14]. Pinto et al. [15] found that adherence to prescribed exercises it is directly correlated to achieving treatment goals and obtaining desired increases in physical function. Non-compliance to home exercise programs is common with over 50% of the patients only partially adhering to their program and over 15% not adhering at all [16]. The reasons vary and include exercises are too boring, the patient did not remember how to do them correctly or completely forgot about them [16]. No information on the effect of pain with exercising on adherence to home programs is available, but the trajectory of adherence seems to be related to baseline pain [17]. Written exercise instructions are often given to the patient to remind him or her of the exercise. This simple strategy has been shown to be effective in promoting adherence to exercise [18] and improve compliance to home-based exercises [19]. Unfortunately, depending on the age of the individual, some patients do not remember exercises effectively after a single teaching session, even if they were given an instructional exercise pamphlet [20].

Previous studies [21–23] have assessed retention and quality of exercises by using pre-defined clinical observation protocols. These protocols assess the amount of cueing needed, and control, coordination and rhythm of the exercise [22] or specific physical components of each exercise [21, 23]. However, those can be subjective and might not be able to detect subtle changes in exercise performance. In this study we used a marker-based motion capture system that yields objective quantitative data [24].

The purpose of this study was to determine whether older adults can accurately replicate physical therapy exercises for the knee, when those exercises are performed away from the direct supervision of a licensed physical therapist. We chose this particular age because it reflects the time in life where osteoarthritis in the knee becomes more prevalent and the need for primary total knee arthroplasty more frequent. We hypothesized that older adults are unable to accurately replicate the aforementioned physical therapy exercises.

## Methods

### Participants

A small convenience sample of 19 healthy volunteers (9 males and 10 females, age [y] 63.1 ± 8.6, mass [kg] 76.3 ± 14.7, height [m] 1.7 ± 0.1) participated in this study (Table 1). No a priori power analyses were performed before conducting the study. These individuals were recruited by word of mouth. Inclusion criteria were > age 45 and no injury to the legs within the past 6 months. IRB approval was obtained from the appropriate institutional review board.

**Table 1:** Participants Demographics

Characteristics	Mean (SD)
**Gender (males/females)**	9/10
**Age (years)**	63 (9)
**Height (cm)**	169 (10)
**Mass (Kg)**	76 (15)
**Physical Activity (days)**	4.3 (1)
**Compliance (days)**	4.8 (2)
**Dominance (R/L)**	14/5

Physical activity represents the subject’s response to the question: How many days each week do you exercise? Compliance indicates the subject’s response to the question: How many days did you perform the prescribed exercises in the past week? Participants were asked to perform exercise 5 times each, 5 days/week

### Data collection

The participants wore compression shorts or biker pants. All data collection occurred in the university biomechanics laboratory. To obtain kinematic data, 36 reflective markers were attached on participant’s lower extremity over the following anatomical landmarks: anterior/posterior superior iliac spines, top of the iliac crest, medial/lateral epicondyles of the femur, medial/lateral malleolus, dorsal aspect of the heads of 1st and fifth metatarsals, lateral aspect of calcaneus. Rigid clusters of 4 markers each were attached to the distal-lateral thigh and lower leg. Twelve high speed video cameras (100 Hz; Qualisys, Inc., Gothenburg, Sweden) were used to track the 3D positions of each reflective marker while the participants perform the rehabilitative exercises.

**Fig. 1:**
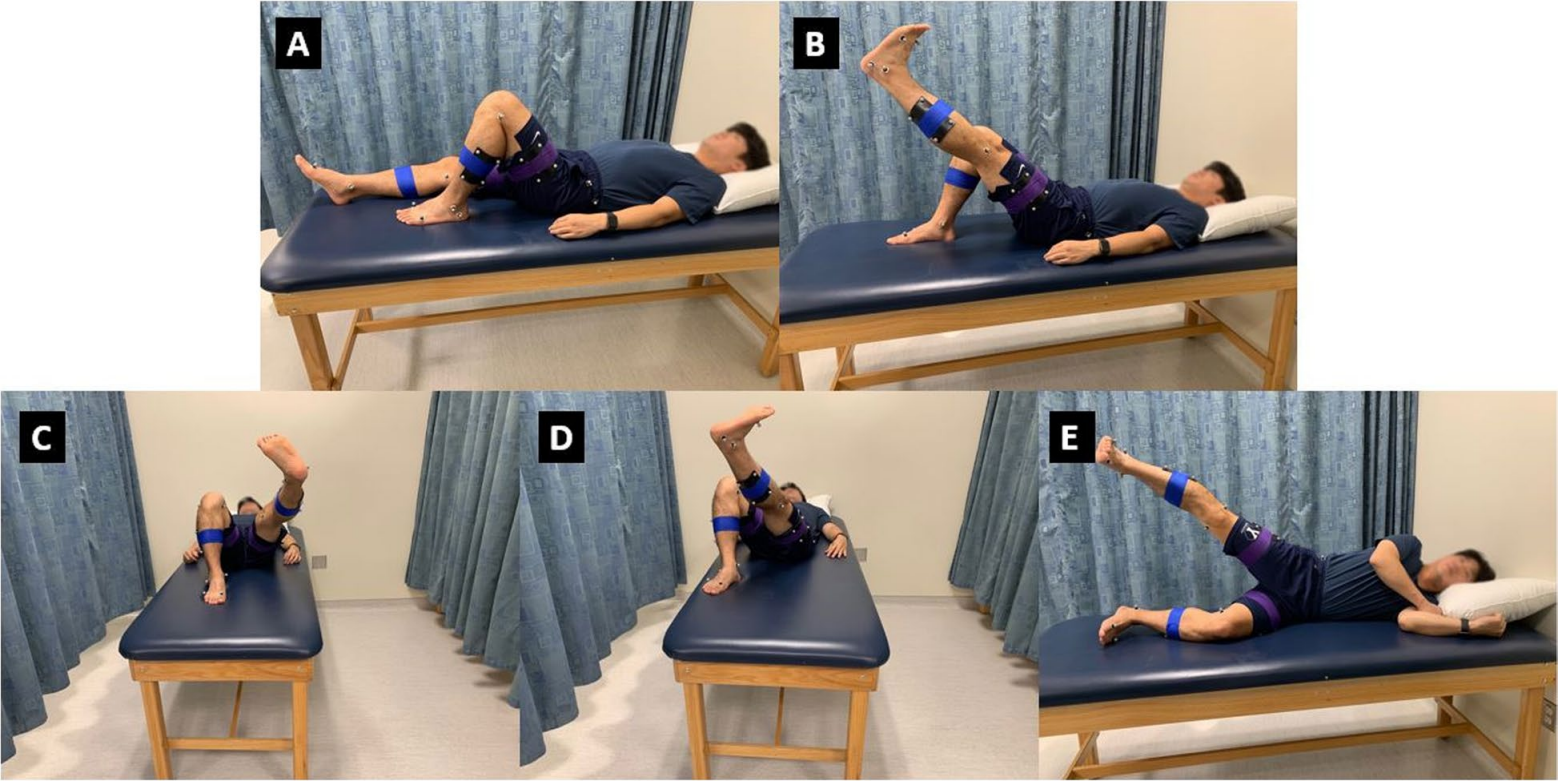
Images of all exercises. **A**: knee flexion exercise; **B**: straight leg raise; **C**: “V out” exercise; **D**: “V in” exercise; **E**: “Jane Fonda” exercise

Each participant was instructed by the same physical therapist. No particular protocol was used; the goal was to have the participant perform the exercises correctly. Different modes of teaching were employed, such as demonstration, verbal explanation, and manual guidance. All exercises (see Fig. 1) are open chain exercises that are meant to promote neuromuscular control and strength of the intended muscle group [25]. They were selected because they are commonly used as part of a knee rehabilitation protocol and do not require special equipment.

The participants were positioned on a treatment table with a pillow placed under the head. The sequence of exercise was always the same though the starting side (right or left) was randomized. Each exercise was performed 5 times on each side. The first exercise was called ‘knee flexion’. While in supine position the participant was told to bend the knee by sliding the heel as close to the buttocks as possible, then return to the original position. This basic exercise was chosen as a control movement, in that there should not be a difference in its performance between the two visits. The second exercise was called ‘straight leg raise’ (SLR). A rolled-up hand towel was placed underneath the knee of the exercising leg. The participant was told to push the knee into the towel and lift off the heel in order to produce terminal extension. The cue to the participant was to then “draw the toes toward the nose and let the heel be the guide of the raising leg motion”. It was explained that when the heel, not the knee, guides the motion one is less inclined to flex the knee. Knee extension was to be maintained throughout the exercise. The contralateral knee was flexed to about 90° with the foot resting on the table. Once the knee of the raising leg was at the same height of the contralateral knee the raising leg could be lowered again. We chose to limit hip flexion to avoid active and passive insufficiency of the rectus femoris and the hamstring muscles, respectively. The muscles were to relax after each exercise. This exercise is typically chosen to focus on and strengthen terminal knee extension. The third exercise was called the “V”. This exercise was chosen because it is more challenging. It incorporates hip rotation and as such might be more demanding. Hip muscles, especially the gluteal muscles, are important pelvic stabilizers that can influence lower limb alignment [26]. Their activation depends partially on the direction of hip rotation [25, 26]. The “V” exercise was separated into two parts. During the first part the straight leg was internally rotated at the hip and, while performing hip flexion/abduction, the heel was “writing” the one side of the “V”. For the second part of this exercise the leg was externally rotated at the hip and, while performing a SLR into adduction, the heel was writing the other side of the “V”. This exercise focuses on the lateral (hip abductors and internal rotators) [27] and medial muscle groups (vastus medialis oblique and hip adductors) [28] of the lower extremity kinematic chain, respectively. It is crucial to perform this exercise with the hip rotated in the correct direction in order to recruit the targeted muscle groups [27, 28]. This was explained to the participant. The fourth exercise was called the ‘Jane Fonda exercise’ and was performed in side lying position. It required the participant to abduct the hip in the frontal plane with neutral hip rotation. Care was taken to point out that the abduction was to happen in the plane of the body and the hip was not to be flexed. This single-plane open chain exercise promotes neuromuscular control and strength of the gluteus medius when performed properly [29].

All participants received feedback in form of tactile stimuli and verbal cues during the instructional phase from the same person. This usually took up to three repetitions per exercise. During the first data collection phase the physical therapist observed exercise performance after the instructions were given and, when necessary, corrected the movements by guiding the participant verbally. At the end of the first data collection day each participant received an instructional pamphlet with pictures and the recognizable names of the exercises. Each participant was encouraged to perform the exercises every day for one week, five repetitions each. The instructions were meant to reflect the routine of clinical practice. The participants were informed that they were expected to perform the same exercises again one week later without instructions.

One week later, the participants returned to the aforementioned biomechanics laboratory. Reflective markers were attached to the bony landmarks in the same arrangement as before and the participants were asked to perform the same exercises, five repetitions each. No cues, other than the names of the exercises, were given. “Correct performance” was defined by the following variables: absolute difference in internal/external hip rotation of less than 10° and knee extension difference by more than 5° during the second visit compared to the first.

### Data acquisition and processing

The 3D trajectories for each reflective marker were tracked using Qualisys software (more info likely needed here), and then exported to Visual 3D software (C-Motion, Germantown, MD, USA) in order to calculate the 3D hip and knee joint angles. Before the joint angles were calculated, the marker trajectories were smoothed using a fourth-order, low-pass Butterworth filter with a cutoff frequency of 6 Hz and time normalized in percentage. To calculate hip joint internal and external rotation, during the “V” exercises, we used a foot segment and lab coordinate as reference segments to eliminate errors from marker trajectories on the hip. The kinematic variables that were compared between the first and second data collection sessions are described in Table 2. Specifically, for the knee flexion exercise, maximum knee extension was used to define the beginning and end of each repetition. We used maximum hip extension to define the beginning and end of each repetition for the “V” exercises and SLR. Similarly, we used maximum hip adduction to define the beginning and end of each repetition for the “Jane Fonda” exercise. Peak and average joint angles were calculated for this study. To calculate peak angle, the maximum was identified for each repetition and then these values were averaged across the five trials for each participant. To calculate the average angle, the mean joint angle across the entire duration of each repetition was calculated and then averaged across the five trials for each participant. The angles of interest for each exercise were: knee flexion average (for SLR, and “V” and “Jane Fonda” exercises), hip internal/external rotation average (for the “V” exercise), hip flexion average (for the “Jane Fonda” exercise), and peak knee flexion/extension (for the knee flexion and SLR exercises). Only data for the dominant leg were analyzed. The dominance was identified as the limb that participants use when they kick a ball.

**Table 2:** Hip and knee joint angles measured during the 1st and 2nd. Several joint angles significant differed between the two visits, indicating that subjects were unable to consistently replicate rehabilitation exercises seven days after being taught by a physical therapist

	Exercises	Dependent Variable	Joint Angle, Mean (SD)		***p*** value	MDC (°)
			1st visit	2nd visit		
**Peak Angle**	**Knee Flexion**	Knee Flexion/Extension^a^	117.3 (12.2)	116.1 (14.3)	0.69	26.9
	**SLR**		−7.7 (5.5)	−10.8 (6.0)	0.02*	10.9
**Average Angle**	**SLR**	Knee Flexion	2.1 (4.6)	6.4 (4.9)	< 0.01*	10.6
	**V “Out”**	Knee Flexion	4.4 (5.6)	7.3 (5.2)	0.11	6.2
		Hip IR (+)/ER(−)	12.4 (19.0)	−16.3 (29.4)	< 0.01*	60.6
	**V “In”**	Knee Flexion	3.4 (3.3)	8.4 (3.8)	< 0.01*	14.7
		Hip IR (+)/ER(−)	−46.1 (12.5)	−12.5 (30.2)	< 0.01*	64.0
	**“Jane Fonda”**	Knee Flexion	1.6 (5.8)	5.3 (9.5)	0.07	9.1
		Hip Flexion	8.3 (9.4)	13.0 (12.8)	0.13	19.4

Peak angles represent the peak within each trial. Average angles represent the average angle across the entire trial. ^a^Zero degrees represent anatomical position; positive knee angles indicate knee flexion and negative values indicate hyperextension; zero degrees represents anatomical position. * significant difference between 1st and 2nd visit. *SD* standard deviation, *SLR* straight leg raise, *MDC* minimal detectable change

### Statistical analysis

Assumptions for paired t-tests were met: participants were independent from one another, data were normally distributed, outliers were removed, and the dependent variables were continuous. Consequently, paired t-tests were used to compare the [1] peak angle, and [2] average angle throughout the entire trial, with and without direct supervision of a physical therapist. Speaking generally, we chose to consider the peak and average (throughout the entire trial) angles because they each represent different qualities of motion: peak angles reflect range of motion, while average angles potentially represent other movement characteristics (e.g., muscular strength or variability in neuromuscular activation). Significance was set a priori at *p* < .05. All statistical analyses were conducted using JMP Pro 15 (SAS Institute Inc., Cary, NC). To aid the clinical interpretation of between-day differences in the joint angles, the minimal detectable changes were calculated as previously described [30].

A mean of peak angles and the averaged angles throughout each variable were used to identify overall angle throughout each cycle. A repeated measures t-test was used to compare the performance of the same exercises between with and without supervision of a physical therapist.

## Results

Between the first and second data collection sessions the participants reported that they performed the exercises at home an average of 4.83 ± 1.75 days a week (Table 1). Participants also reported that they were involved in other exercises, such as walking, biking, stretching (Table 1).

Mean peak joint angles and average angles are shown in Table 2. As expected, there was no difference in mean peak knee flexion angles during the knee flexion exercise between the two visits (Table 2).

The “V” exercise exhibited the largest changes in kinematics between visits and between participants, with second visits demonstrating standard deviations up to twice the magnitude of the mean. On average, the participants used almost 30° less hip internal rotation and over 30° less hip external rotation during “V out” (*p* = 0.0003) and “V in” (*p* < 0.0001) exercises, respectively, at the second visit compared to the first visit. During SLR the participants exhibited almost 3° less peak knee extension at the second visit compared to the first visit (*p* = 0.024). In addition, they demonstrated 4.3° and 5° greater average knee flexion angle (i.e. less extension) throughout the 5 repetitions during SLR (*p* = 0.001) and “V out” (*p* = 0.0001) exercises at the 2nd visit compared to the 1st visit, respectively. There were no differences in knee flexion (*p* = 0.069) and hip flexion (*p* = 0.125) averaged angles during “Jane Fonda” exercise between visits.

**Fig. 2:**
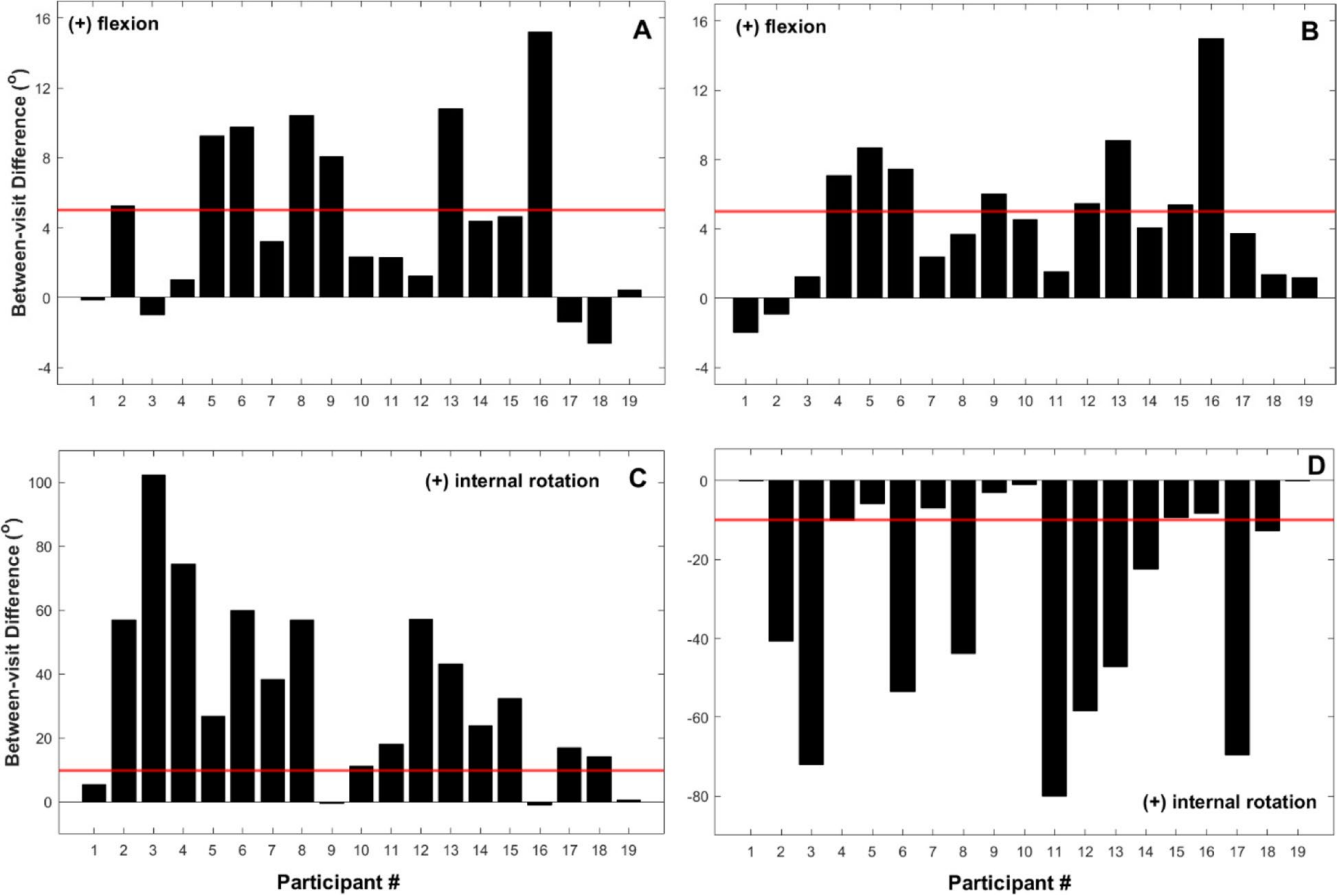
**A** Between-visit differences (second visit minus first visit) in average knee joint angle during the single-leg raise exercise. **B-C** Between-visit differences in knee flexion and hip rotation angles, respectively, during the “V in” exercise. **D** Between-visit differences in hip rotational exercises during the “V out” exercise. The red lines represent the magnitudes of error (°) that make a clinically significant difference; between-visit differences between the zero and red lines indicate participants who performed the exercise correctly

**Fig. 3:**
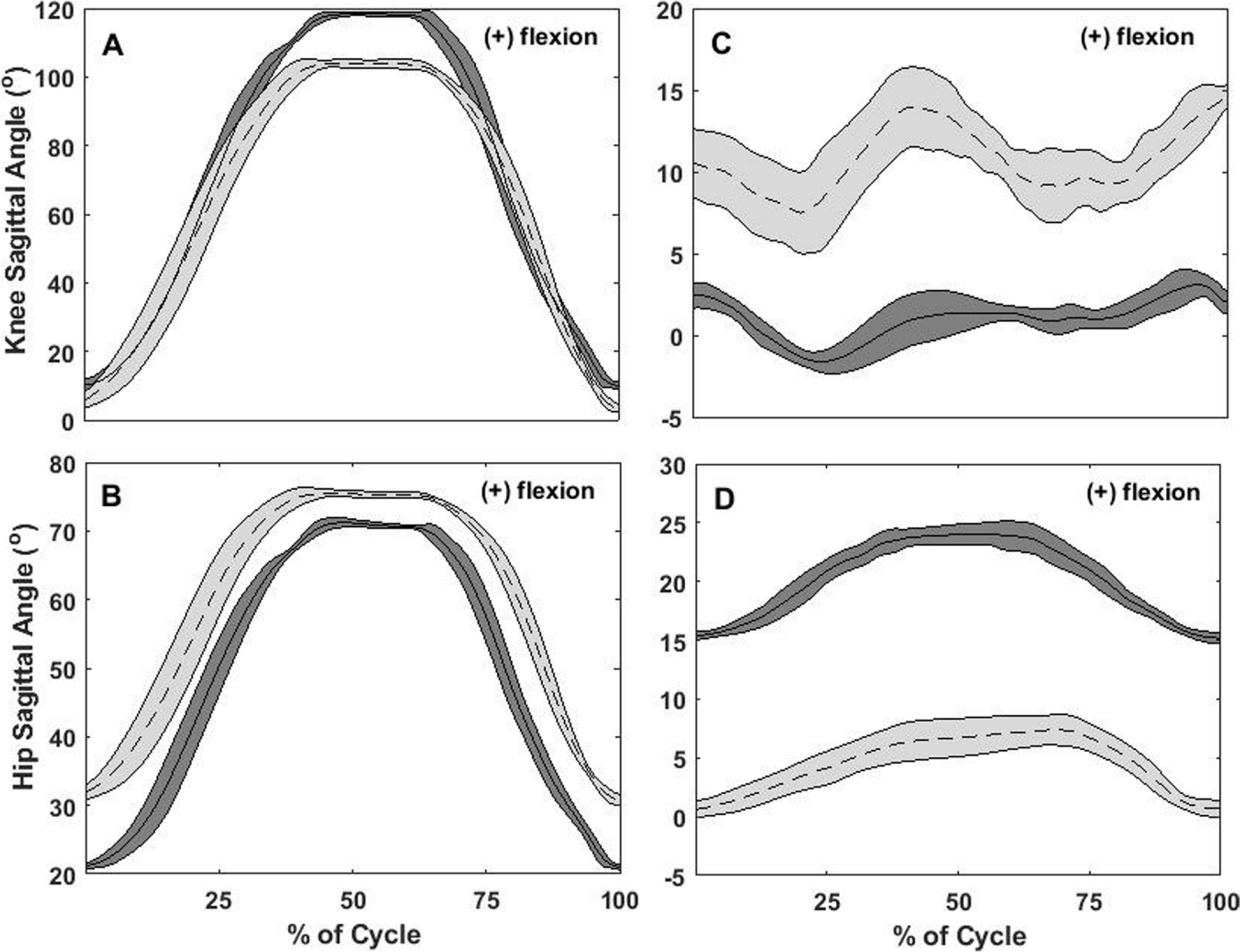
Graphs of the hip and knee angles during knee flexion, SLR, and “Jane Fonda” exercises. The graphs represent only participant 6. **A**: knee angles in the sagittal plane (flexion/extension) and **B**: hip angle in the transverse plane (rotation) during the knee flexion exercise; **C**: knee angles in the sagittal plane (flexion/extension) during SLR and **D**: hip angle in the sagittal plane (flexion/extension) during the “Jane Fonda” exercise). Solid lines represent the average of 5 repetitions for each exercise at the first visit while dotted lines represent the same for the second visit; the shaded gray area around each line represents standard deviation of the 5 repetitions; X-axis: time normalized in percentage

**Fig. 4:**
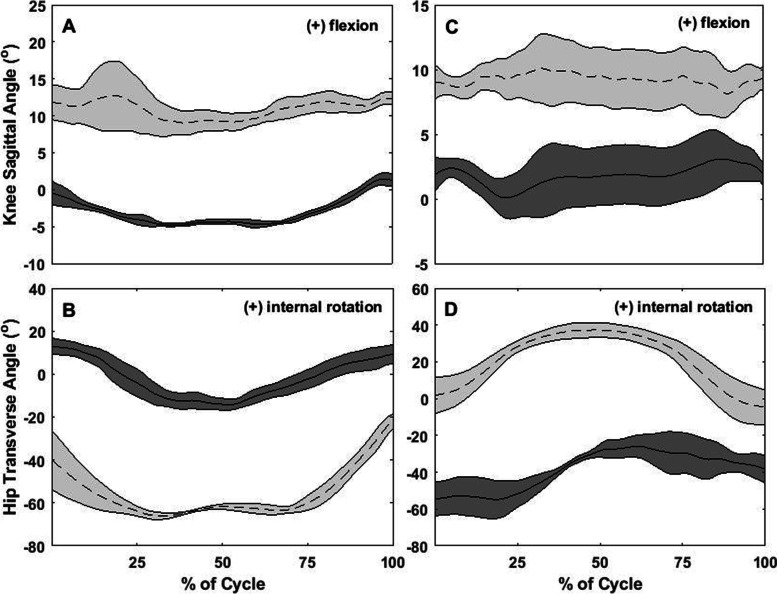
Graphs of the hip and knee angles during “V out” and “V in” exercise. The graphs represent only participant 6. **A**: knee angles in the sagittal plane (flexion/extension) and **B**: hip angle in the transverse plane (rotation) during the “V out” exercise; **C**: knee angles in the sagittal plane (flexion/extension) and **D**: hip angle in the transverse plane (rotation) during the “V in” exercise. Solid lines represent the average of 5 repetitions for each exercise at the first visit while dotted lines represent the same for the second visit; the shaded gray area around each line represents standard deviation of the 5 repetitions; X-axis: time normalized in percentage

**Fig. 5:**
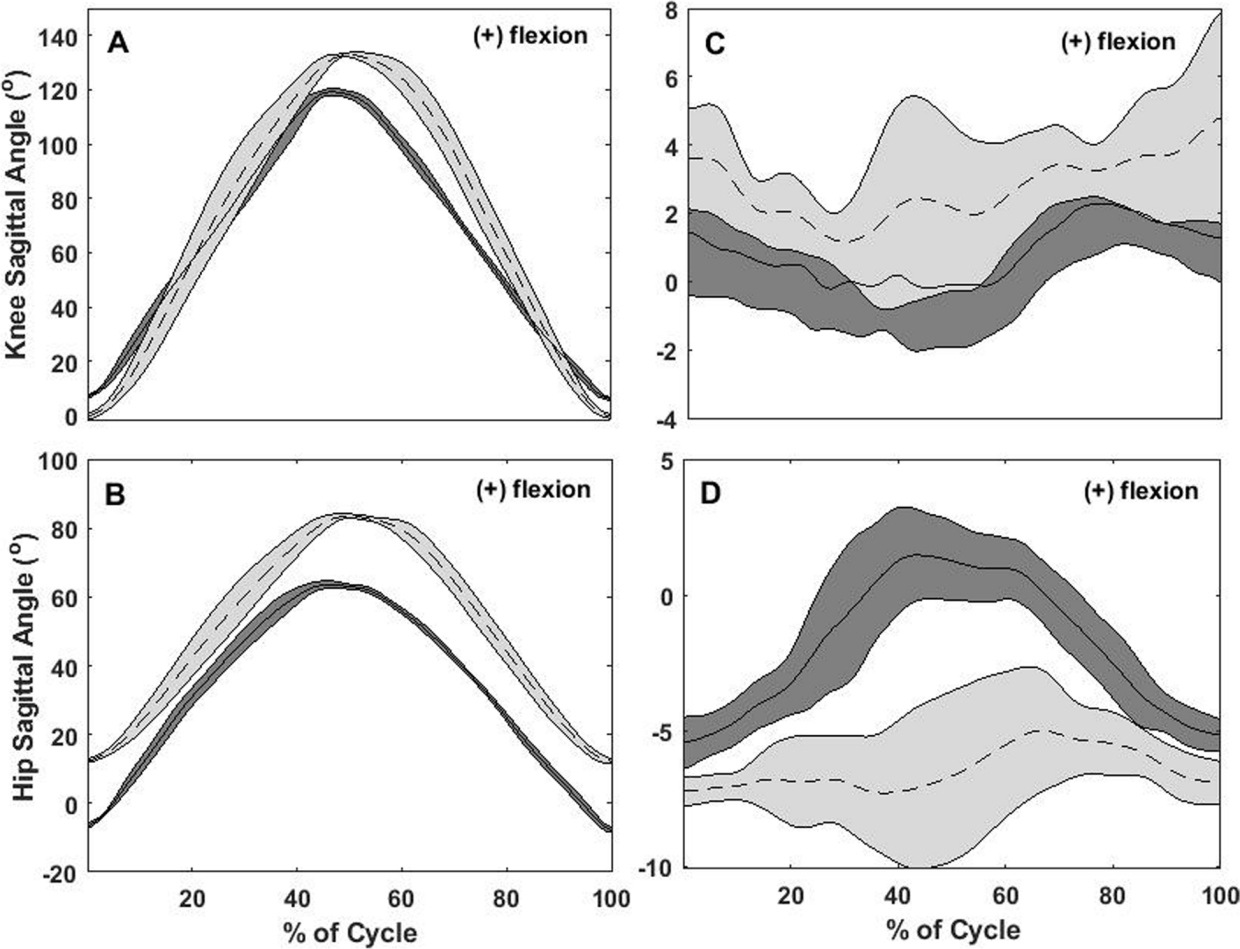
Graphs describing no difference between visits for the flexion exercise, SLR, and “Jane Fonda” exercise. **A**: knee angles in the sagittal plane (flexion/extension) and **B**: hip angle in the transverse plane (rotation) during the knee flexion exercise; **C**: knee angles in the sagittal plane (flexion/extension) during SLR and **D**: hip angle in the sagittal plane (flexion/extension) during the “Jane Fonda” exercise). Solid lines represent the average of 5 repetitions for each exercise at the first visit while dotted lines represent the same for the second visit; the shaded gray area around each line represents standard deviation of the 5 repetitions; X-axis: time normalized in percentage

**Fig. 6:**
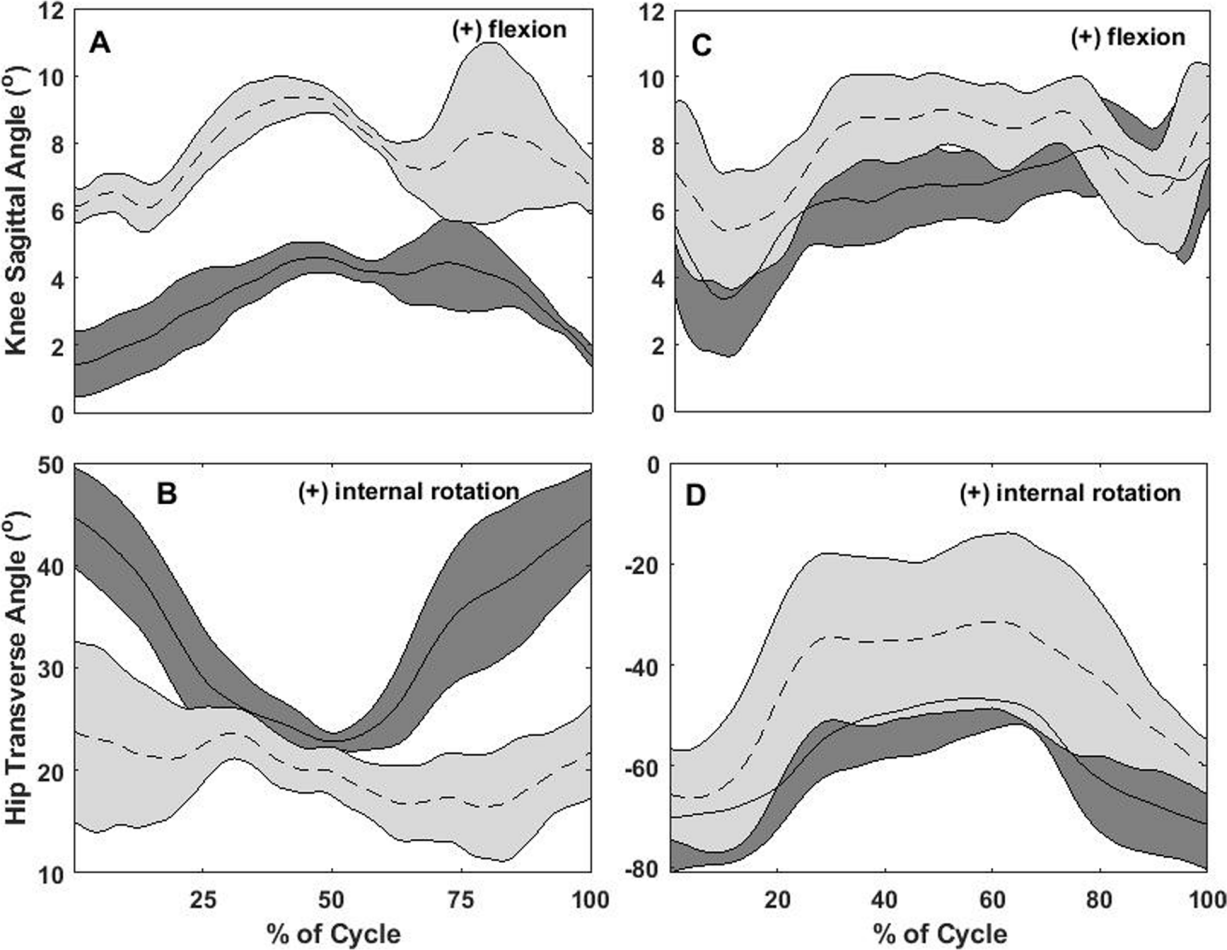
Graphs describing no difference between visits for the “V out” and “V in” exercises. **A**: knee angles in the sagittal plane (flexion/extension) and **B**: hip angle in the transverse plane (rotation) during the “V out” exercise; **C:** knee angles in the sagittal plane (flexion/extension) and **D**: hip angle in the transverse plane (rotation) during the “V in” exercise. Solid lines represent the average of 5 repetitions for each exercise at the first visit while dotted lines represent the same for the second visit; the shaded gray area around each line represents standard deviation of the 5 repetitions; X-axis: time normalized in percentage

Between-day differences for all participants, for all of the kinematic variables, are depicted in Fig. 2. Figures 3, 4, 5 and 6 show two different kinds of participants. Figures 3 and 4 depict ensemble means (averaged across all five repetitions) for kinematic variables for one participant who exhibited relatively great changes between Days 1 and 2. Figures 5 and 6 show ensemble means (averaged across all five repetitions) for kinematic variables for a participant who exhibited minimal changes between Days 1 and 2.

## Discussion

The purpose of this study was to determine whether healthy middle-aged and older adults can accurately replicate post TKA physical therapy exercises one week after learning how to perform the exercises from a licensed physical therapist. In order to answer this query we obtained objective and quantitative data by using a marker-based motion capture system. Our results show that not one of the participants was able to accurately replicate all the aforementioned physical therapy exercises (Fig. 2) and that exercise performance (as determined by hip and knee joint kinematics) declined significantly one week after instruction. This decline occurred although every participant was given an instructional exercise sheet.

More specifically, the most frequent, obvious and severe change in exercise performance within one week of instruction occurred with the “V” exercises. In order to target the lateral hip muscles the “V out” phase needs to be performed with hip internal rotation [27]. However, during the second visit the hip was, on average, rotated 232% more into external rotation during this exercise compared to the first visit. Consequently, it was performed using the anterior muscle chain (quadriceps) and not, as intended, the abductors. Eleven participants performed this exercise incorrectly. The “V in” phase was performed with 73% less hip external rotation in the second visit compared to the first, therefore also promoting the anterior muscle chain, and not, as intended, the adductors [28]. Fifteen participants performed this exercise incorrectly. Throughout all exercises that require terminal knee extension the knee was, on average, held in more flexion during the second visit. This indicates that terminal extension was, on average, not maintained. Full extension is an important part of the exercises because it is the position in which the oblique head of the vastus medialis is best activated [31].

Standard deviation was up to twice as high during the second visit for hip rotation during the “V in” phase compared to the first visit. With the exception of one variable (knee flexion during the “V out” exercise) all standard deviations were higher during the second visit. This could be a reason why some of the differences in joint angles between visits are not statistically significant. More importantly the greater variability between participants during the second visit indicates that some participants retained the exercises better than others, suggesting that the exercise benefits, i.e. strengthening of the targeted muscle groups, also varied. A similar phenomenon was reported by Faber et al. [23] who compared shoulder exercise form and quality during time of exercise instruction and two weeks thereafter. Exercise form was determined using a pre-defined clinical observation protocol, and exercise quality was defined by the muscle’s time under tension. They found that only 13 of the 29 participants used correct exercise form. In addition, the standard deviation of the exercise quality, i.e. time under tension, was almost four times as high during the second visit compared to the first. The authors surmised that this variability in quality reflects the fact that some participants received a 50% higher exercise dose and others a 40% lower exercise dose than intended [23].

According to our definition of ‘correct performance’ no participant was able to perform all exercises correctly in our study (Fig. 2). Hip flexion during the “Jane Fonda” exercise was also considered as a potential source for incorrect performance, but this variable was not different between the two visits. Six of the 19 participants performed only one exercise incorrectly, while one participant made a mistake on every exercise. Only four participants exhibited the correct (external) hip rotation amount and angle during the “V in” exercise.

As expected, there was no difference in mean peak knee flexion angles during the knee flexion exercise between the two visits (117° and 116°, respectively). This exercise was used as reference exercise, meaning, no change was expected to occur over one week. Had there been a disagreement between the data collected on the first visit and the ones collected on the second visit it could have indicated that our methodology was not sound.

We are aware that not every participant, and patient, responds to and retains home exercise instructions the same. One could argue that five exercises are too many to recall. We decided to use 5 exercises based on the findings by Henry et al. [22]. They found that there is no difference in quality of performance between executing two or five exercises as measured by the amount of cueing needed, and control, coordination, and rhythm of the exercise. Others [21] have used the same amount of exercises in their studies that test their recall.

While our study’s methodology was strengthened by its use of objective and validated outcome measures, the fact that our participants were not actual patients remains a limitation. A strong barrier for adherence to a home exercise program is pain when performing the exercises [13]. We did not collect data on knee pain, so this barrier was not, or at least not strongly, present. Additionally, the correct execution of the exercises might not have presented a priority to our participants, as it might have been to patients after knee surgery. On the other hand, our participants were probably able to perform the exercises better because they were not actual patients. Either way, our results cannot be generalized to actual patients. Another limitation is the low number of exercise repetitions we used in this study (i.e., five). A more pragmatic model would have been to have the participant perform three sets of 10 exercises to induce a more pronounced learning effect. The small sample size is another limitation of the current study, which might have caused a type 1 error. Therefore, future studies with a larger sample size are needed to strengthen the results of the current study. Lastly, our study exclusively measured quantitative data and did not measure perception of exercise difficulty or complexity which could have had an influence on the quality of exercise performance.

## Conclusion

Thorough and individual instructions into lower extremity exercises and an instructional pamphlet are not enough to ensure proper execution of the home exercise program. Other methods need to be explored to get the greatest possible benefit from home exercises.

## Data Availability

The datasets supporting the conclusions of this article are included within the article. The raw data can be requested from the corresponding author.

## Abbreviations

TKA: Total Knee Arthroplasty
SLR: Straight Leg Raise
